# Pharmacognostical and Phytochemical Studies and Biological Activity of *Curculigo latifolia* Plant Organs for Natural Skin-Whitening Compound Candidate

**DOI:** 10.1155/2023/5785259

**Published:** 2023-10-04

**Authors:** Syamsu Nur, Heri Setiawan, Muhammad Hanafi, Berna Elya

**Affiliations:** ^1^Department of Phytochemistry and Pharmacognosy, Faculty of Pharmacy, University of Indonesia, Depok 16424, Indonesia; ^2^Department of Pharmaceutical Chemistry, Almarisah Madani University, Makassar 90245, Indonesia; ^3^Department of Pharmacology, Faculty of Pharmacy, University of Indonesia, Depok 16424, Indonesia; ^4^Indonesian Institute of Sciences (National Research and Innovation Agency (BRIN)), Jakarta 15314, Indonesia; ^5^Department of Phytochemistry, Faculty of Pharmacy, Pancasila University, South Jakarta 12640, Indonesia

## Abstract

*Curculigo latifolia* (family Amaryllidaceae) is used empirically for medicinal purposes. It is distributed throughout Asian countries, especially Indonesia. This study aimed at standardizing the *C. latifolia* plant, analyzing its phytochemical profile, and evaluating its pharmacological effects. The powder from each organ (root, stem, and leaves) was standardized organoleptically and microscopically. Samples were extracted by graded maceration using hexane, ethyl acetate, and ethanol. The extracts were determined for total phenolic content (TPC) and total flavonoid content (TFC). Antioxidant (radical scavenging and metal ion reduction) and antityrosinase activities were determined by spectrophotometric methods. Extracts were analysed for phytochemical profiles by LC-ESI-MS. The highest TPC and TFC were found in the ethanolic extract of the root organ (68.63 ± 2.97 mg GAE/g) and the ethyl acetate extract of the stem (14.33 ± 0.71 mg QE/g extract). High antioxidant activities were found in the ethanolic root extract (20.42 ± 0.33 *µ*g/mL) and ethanolic stem extract (45.65 ± 0.77 *µ*g/mL) by DPPH• and NO• assays, respectively. The ion reduction activity (by CUPRAC assay) was most significant in the ethyl acetate stem extract (390.42 ± 14.49 *µ*mol GAEAC/g extract). Ethanolic root extract was the most active in inhibiting tyrosinase (IC_50_ value of 108.5 *µ*g/mL). The correlation matrix between TPC and antioxidant activities showed a moderate to robust correlation, whereas the TPC and antityrosinase activity showed a robust correlation. The TFC and antioxidant or antityrosinase activities showed a weak to moderate correlation. The LC-ESI-MS data identified major phenols in the active extracts, including methyl 3-hydroxy-4-methoxy-benzoate, quercetin, 4-O-caffeoylquinic acid-1, and curculigoside. Overall, this study suggests that extracts from the *C. latifolia* plant offer potent antioxidant and antityrosinase activities, allowing them to be used as natural antioxidants and candidates for skin-lightening compounds.

## 1. Introduction

The skin is the outermost layer of the human body. Excessive sun exposure stimulates the formation of melanin pigments. Melanin is the main pigment responsible for human skin, hair, and eye pigmentation. Melanocytes produce melanin through melanogenesis. Melanogenesis and skin pigmentation are the main photoprotective factors in response to harmful UV radiation from sun exposure and skin photocarcinogenesis. Melanin loss and irregular depigmentation loss can represent serious aesthetic and dermatological problems on the face in humans [1–3]. Conversely, increased melanin synthesis and excessive accumulation of melanin pigment can cause various skin diseases such as acanthosis nigricans, cervical poikiloderma, melasma, periorbital hyperpigmentation, lentigines, neurodegeneration associated with Parkinson's disease, skin aging, and risk of skin cancer [3, 4].

During melanogenesis in the skin, tyrosinase is an important enzyme in melanin synthesis. Tyrosinase is also a multifunctional copper-containing metalloenzyme with binuclear copper ions and acts as a reaction accelerator in melanin synthesis. In addition, the obvious effect of melanogenesis due to the hyper-reaction of tyrosinase is the formation of hyperpigmentation of the skin [5, 6]. Tyrosinase is the main cause of unwanted tanning due to the overproduction of melanin. Therefore, control of enzyme activity by tyrosinase inhibitors is important to treat hyperpigmentation, especially in skin tissues, to keep the skin white and glowing. Many effective inhibitors have been identified and developed in medical and cosmetic skin-lightening products. However, in medicine, only a few compounds are known to be potent and safe tyrosinase inhibitors [3, 7].

The search for bioactive compounds provides relatively safe biological activity through plant development. Since plants are a rich source of bioactive chemicals, most of which are free from harmful side effects, there is an increasing interest in finding skin-lightening products from natural sources. One of the plants that can be developed as a candidate for skin-lightening compounds is *Curculigo latifolia*. *C. latifolia* or *Molineria latifolia* (Dryand. ex. W.T. Aiton) is a plant of the Amaryllidaceae or Hypoxidaceae family, widely distributed in China, Japan, Nepal, Malaya, India, Australia, and Africa. The original distribution of this plant came from India, Myanmar, Thailand, the Philippines, the Malaysian Peninsula, Singapore, and especially Indonesia, such as Sumatra, the Lingga Islands, the Bangka Island, Borneo, Java, and Celebes [8–10]. In different regions of Indonesia, *C. latifolia* plants are also known as marasai, lumbah, marasi, congkok, and doyo. Parts of the *C. latifolia* plant, such as leaves, roots, fruits, and flowers, have been empirically used by the community as a traditional medicine to treat bloody urine, wounds, constipation, hemorrhoids, and fever and as a source of energy. Additionally, people in Kalimantan, Indonesia, use the leaves and roots of *C. latifolia* to cure jaundice or hepatitis B [8, 10].

Scientifically, *C. latifolia* has been reported to have antioxidant [9–13], ultraviolet protection [14], antidiabetic [8, 10, 11, 15], and antibacterial [9] effects. Although scientific extracts from plants have not been described as antityrosinase, their bioactivity as antioxidants and UV protection establish a pathophysiological relationship with their activity as antityrosinase, making it possible to develop them as candidates for skin-lightening ingredients. The bioactivity of *C. latifolia* is strongly supported by the content of secondary metabolites found in these plants. Some information showed that the roots of *C. latifolia* contain phenolic compounds such as phloridzin, pomiferin, scandenin, and mundulone [12]. The study by Umar et al. [10] also reported that *C. latifolia* contains phenolic glycosides such as orchioside derivatives, curculigoside derivatives, and triterpenes (cycloartane) groups, namely, curculigosaponin derivatives. Additional information has also been reported that the roots of the *C. latifolia* plant contain flavonoid derivatives such as isorhamnetin, apigenin, hesperetin, and quercetin [16]. The leaves have been reported to contain phenolic glycosides and saponin derivatives (cycloartane triterpenes). Other studies have also revealed that neoculin and curculin compounds have been isolated from *C. latifolia* fruit and showed different pharmacological activities [17, 18].

The presence of phenolic glycosides and flavonoid derivatives in *C. latifolia* has been previously reported so that bioactivity as an antityrosinase can be developed. Several studies have shown that the presence of phenolic and flavonoid derivatives in natural products significantly inhibits tyrosinase activity. The number of OH groups in phenolic and phenolic hydroxyl groups in the A and B rings of flavonoids can enhance the inhibitory effect on tyrosinase [19]. *C. latifolia* plants are a rich source of phenolic compounds and flavonoid derivatives, allowing the development of active skin-lightening ingredients.

Current research aims at investigating the biological activities of the *C. latifolia* plant organs, including their antioxidant and tyrosinase inhibitory activities. Microscopic characterization and phytochemical screening of plant parts were also undertaken. The knowledge gained provides important information for the development of *C. latifolia* as a raw material for medicines and cosmeceuticals.

## 2. Materials and Methods

### 2.1. Chemicals and Instrumentation

2,2-Diphenylpicrylhydrazyl (DPPH), mushroom tyrosinase (CAS: 9002-10-2), 3-(3,4-dihydroxyphenyl)-L-alanine (L-DOPA, CAS: 59-92-7), quercetin, neocuproine, phosphate-buffered saline (PBS), cupric chloride dihydrate, and sodium nitroprusside were purchased from Sigma-Aldrich (USA). Sulfanilic acid, glacial acetic acid, naphthalene diamine dichloride, and gallic acid were purchased from Merck (Germany). Solvents used were of analytical grade from Merck (Germany). The instrumentations used were UV-Vis spectrophotometer (Shimadzu UV-1900, Japan), microplate reader (GloMax, Promega, UK), rotary vacuum evaporator (Buchi R-300, USA), and microscope (XSG, WF10x).

### 2.2. Plant Material

**Figure 1:**
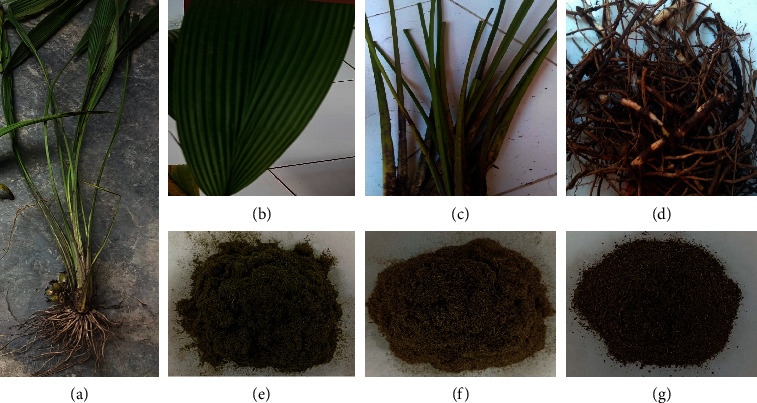
Plant organs of *C. latifolia. C. latifolia* whole plant (a), leaf organ (b), stem organ (c), root organ (d), leaf powder (e), stem powder (f), and root powder (g).

The plant parts used in this study were the roots, stems, and leaves (Figure 1) of *Curculigo latifolia*. They were obtained from the Indonesian Medicinal and Aromatic Crops Research Institute (IMACRI), Menteng, Bogor Baru District, Bogor City, West Java (6°57′70.2″S; 106°78′62.5″7E altitude 15 m). The specimen was collected in August 2021 and identified at the Herbarium Bogoriense Biodiversity Research Organization, Biological Research Center Bogor City, West Java, Indonesia. The process of harvesting *C. latifolia* plants was carried out at 07-08 in the morning before the plants experience active metabolism due to the influence of photosynthesis, so the chemical content of the plants is more stable and does not experience decomposition [20, 21].

### 2.3. Organoleptic and Microscopic Evaluation

Organoleptic evaluation was performed on each organ of *C. latifolia* plants based on sensory parameters including the shape, color, smell, and taste. Microscopic evaluation was performed on *C. latifolia* plant organ powder. Sample powder was placed on a glass object and sprinkled with chloral hydrate as a coloring agent. The samples were then examined for microscopic parameters using a binocular microscope (XSG, WF10x) at a magnification of 400x [22].

### 2.4. Sample Extraction

The roots, stems, and leaves of the *C. latifolia* plant were dried and powdered, followed by gradual maceration using various solvents with different polarity levels [23]. In the present study, each plant organ (250 g) was soaked with *n*-hexane at a ratio of 1 : 10 (powder (g): solvent (mL). The maceration was carried out for 1 × 24 hours at room temperature; thereafter, the macerated sample was filtered. The residue was remacerated with the same solvent until a clear filtrate was obtained. The residue of each plant part was then macerated with ethyl acetate solvent in the same way as with n-hexane solvent. The extraction process was carried out similarly using 70% ethanol. Each pooled filtrate obtained was evaporated using a rotary vacuum evaporator (Buchi, R-100) to obtain a thick extract.

### 2.5. Phytochemical Profiles

Each extract of *C. latifolia* was identified for the presence of a class of phenolic, flavonoid, tannin, saponin, alkaloid, and terpenoid/steroid compounds using specific reagents [24].

### 2.6. Determination of Total Phenolic Content (TPC) and Total Flavonoid Content (TFC)

Analysis of the TPC and TFC of each organ extract of *C. latifolia* was carried out by colorimetry methods using a visible spectrophotometer (Shimadzu UV-1900, Kyoto, Japan) [25]. In the TPC procedure, an aliquot (0.1% w/v, 0.5 mL) of each extract solution was mixed with 0.4 mL of Folin–Ciocalteu (0.2 M) reagent. The mixture was allowed to stand for 3 minutes, and then, 2 mL of Na_2_CO_3_ 7.5% (w/v) was added. The volume was adjusted to 5 mL by the addition of distilled water. The mixture was then homogenized and incubated for 30 minutes at room temperature. The absorption of the sample solution was measured at 635 nm (spectrophotometer UV-Vis, UV-1900, Kyoto, Japan). The TPC of each extract was calculated using the standard curve equation of gallic acid (2–10 *µ*g/mL). The TPC of each extract was expressed as gallic acid equivalent (mg GAE/g extract).

The procedure for determining TFC was as follows. Extract solution (0.1% w/v, 0.5 mL) was mixed with sodium acetate (1 M, 0.1 mL). The mixture was left for 5 minutes to equilibrate, and then, AlCl_3_ (10% w/v, 0.1 mL) was added. The volume was made up to 5 mL with ethanol and incubated for 5 minutes at room temperature. The absorption was read at 430 nm using a spectrophotometer. The TFC of each extract was calculated using the standard curve equation of quercetin (2–10 *µ*g/mL). The TFC of each extract was equivalent to quercetin (mg QE/g extract).

### 2.7. Determination of Antioxidant Activity

Different in vitro methods were used to determine the antioxidant activity of each extract. Nitric oxide (NO•) and DPPH• radical scavenging assays were carried out to observe the ability of each extract to inhibit the action of radical species, whereas the cupric ion reducing antioxidant capacity (CUPRAC) assay was carried out to observe the ability of each extract to reduce metal ions.

#### 2.7.1. NO Radical Reduction Assay

The NO• radical reduction method was performed based on a reported method by Syamsu et al. [26] with slight modifications. A series of samples (10–1000 *µ*g/mL) were prepared in 0.5 mL of sodium nitroprusside (10 mM in phosphate buffer saline solution). The mixture was then incubated at 27°C for 2.5 hours. The mixture was then added to 1.5 mL Griess reagents which consisted of 750 *µ*L of sulfanilic acid (0.33% b/v in 20% v/v acetic acid glacial) and 750 *µ*L of naphthalene (0.1% b/v). Each mixture was made up to a 5 ml volume with phosphate buffer saline solution and was further incubated for 30 minutes at room temperature. The absorption was measured at 535 nm using a UV-Vis spectrophotometer. The percentage inhibition of NO• radicals was calculated using the following formula:

(1) $$\begin{array}{c} \%\text{ inhibition}=\left(\frac{\text{Abs}.\text{blank}–\text{Abs}.\text{sample}}{\text{Abs}.\text{blank}}\right)\times 100\%. \end{array}$$

The antioxidant activity was expressed by inhibition concentration 50% (IC_50_), i.e., the sample concentration that can reduce NO• radicals by 50%.

#### 2.7.2. DPPH Radical Reduction Assay

The DPPH• radical reduction method was assayed based on a method described by Nur et al. [27] with slight modification. A series of samples (10–1000 *µ*g/mL) was prepared. To each sample was added 1 mL of 0.4 mM DPPH• solution. The volume was made up to 5 mL in ethanol, and the mixture was vortexed to homogenize. The sample solution mixture was incubated for 30 minutes in the dark at room temperature. The absorbance of the sample solution was measured at 516 nm on a UV-Vis spectrophotometer. The percentage inhibition of DPPH• radicals was calculated using the following formula:

(2) $$\begin{array}{c} \%\text{inhibition}=\left(\frac{\text{Abs}.\text{blank}-\text{Abs}.\text{sample}}{\text{Abs}.\text{blank}}\right)\times 100\%. \end{array}$$

#### 2.7.3. Ion Reduction by CUPRAC Assay

The CUPRAC assay was carried out based on [28, 29]. A volume of each extract solution was reacted with 1 mL of CuCl_2_ reagent (10 mM), 1 mL of neocuproine (7.5 mM), and 1 mL of ammonium acetate (1 M). The volume was made up to 5 mL with distilled water. The solution mixture was incubated for 30 minutes, and the absorbance was read at 450 nm by a UV-Vis spectrophotometer. A calibration curve was generated using quercetin standard solution. The antioxidant capacity of each sample was expressed as *µ*M gallic acid equivalent antioxidant capacity of extract (*µ*M GAEAC/g extract). The GAEAC value was calculated using the following formula:

(3) $$\begin{array}{c} \text{GAEAC}=\frac{x\text{ value }X\;\left(1\right./\left.1000\right)X\text{ sample volume }X\text{ dilution factor}}{\text{weight sample }\left(g\right)}. \end{array}$$

### 2.8. Antityrosinase Evaluation Activity

The inhibitory activity against tyrosinase of root, stem, and leaf extracts of *C. latifolia* was determined colorimetrically based on the modification of previous methods [30, 31]. The reaction mixture in a 96-well microplate (Iwaki Pyrex) consisted of 80 *µ*L of phosphate buffer (50 mM, pH 6.5), 40 *µ*L of extract solution (1–500 *µ*g/mL), 40 *µ*L of L-DOPA solution (4 mM), and 40 *µ*L of mushroom tyrosinase solution (75 U/mL). The solution mixture was shaken for 60 seconds and incubated for 30 minutes at 25°C. The absorbance was measured using a microplate reader (Promega GloMax) at 490 nm. The blank solution in phosphate buffer was prepared in the same way as the sample solution. Control samples and blanks were made without the addition of the tyrosinase enzyme. The following equation was used to calculate the percentage of tyrosinase inhibition:

(4) $$\begin{array}{c} \text{inhibition}\left(\%\right)=\frac{\left(A-B\right)-\left(C-D\right)}{\left(A-B\right)}\times 100\%, \end{array}$$

where *A* is the absorbance of the blank solution with enzymes (blank), *B* is the absorbance of the blank solution without enzymes (blank control), *C* is the absorbance of the sample solution with enzymes (sample), and *D* is the absorbance of the sample solution without enzymes (control sample). The IC_50_ value was calculated using a linear regression equation generated from plots of sample concentration versus percentage of inhibition (% inhibition).

### 2.9. The Phenolic Composition of the Active Extract by LC-ESI-MS Study

Analysis of the compounds of extracts was carried out using a liquid chromatography-mass spectroscopy (LC-MS) system using the modified methods in [32, 33]. A Waters Acquity UPLC I-Class equipped with a Xevo G2-XS QToF mass spectrometer with an ESI source (capillary 2 kV, temperature 120°C) was used to generate mass spectra. Sample separation was conducted using an ACQUITY UPLC® BEH C18 (1.7 *µ*m × 2.1 mm × 50 mm). Solvent mixtures used consisted of eluent A (H_2_O + 0.1% formic acid) and eluent B (acetonitrile + 0.1% formic acid), with a ratio of 95 : 5 and 0 : 100. Samples were prepared by dissolving 5 mg of solid, followed by filtering through a 0.22 *µ*m nylon filter. The Sample solution (5 *µ*L) was injected. Mass fragmentation of phenolics was identified using the spectrum database (UNIFI) of organic compounds in the instrument application.

### 2.10. Statistical Analysis

All data were obtained from triplication (*n* = 3) and expressed as mean ± SD. Significant differences between mean values were analysed by one-way analysis of variance (ANOVA, *p* < 0.05) using SPSS 23 version. The TPC and TFC data correlations with antioxidant and antityrosinase activities were analysed using Spearman's correlation by Minitab 20 version.

## 3. Results

### 3.1. Organoleptic and Microscopic Analysis

Each organ of *C. latifolia* generally has different organoleptic properties. In particular, each organ has a distinctive smell. In addition, the root organ tasted bitter compared to other organs. The difference may be due to the levels of phytochemicals in the different organs, leading to different organoleptic results, especially in smell and taste (Table 1).

**Figure 2:**
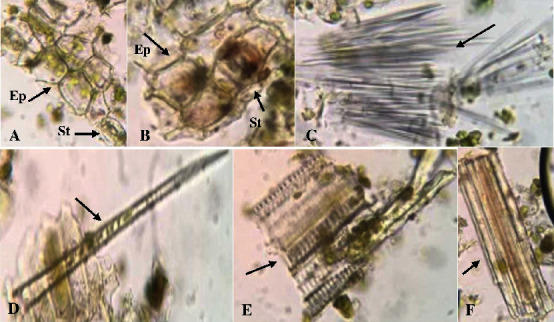
Microscopic analysis of the leaf organ powder from *C. latifolia* plants. Arrows indicate microscopic fragments of leaf organs (magnification 400x). Lower epidermis with stomata (A), upper epidermis with stomata (B), sclerenchyma fibers (C), trichomes (D), bundle sheaths/vessels (E), and fragments of pericyclic fibers with a tortuous wall (F). *Note.* St = stomata and Ep = epidermis.

**Figure 3:**
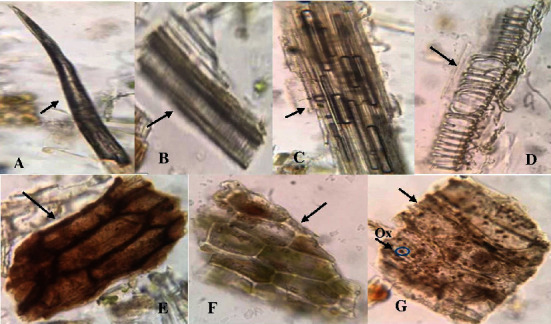
Microscopic analysis of the stem organ powder from *C. latifolia* plants. Arrows indicate microscopic fragments of leaf organs (magnification 400x). Trichomes (A), xylem vessels with helical thickening (B), fiber with crystal fiber (C), fragments of lignified xylem vessels with annular/spiral thickening (D), periderm (E), endosperm containing oil droplets (F), and aleurone grains containing acicular crystal of calcium oxalate (G). *Note*. Ox = oxalate crystals.

**Figure 4:**
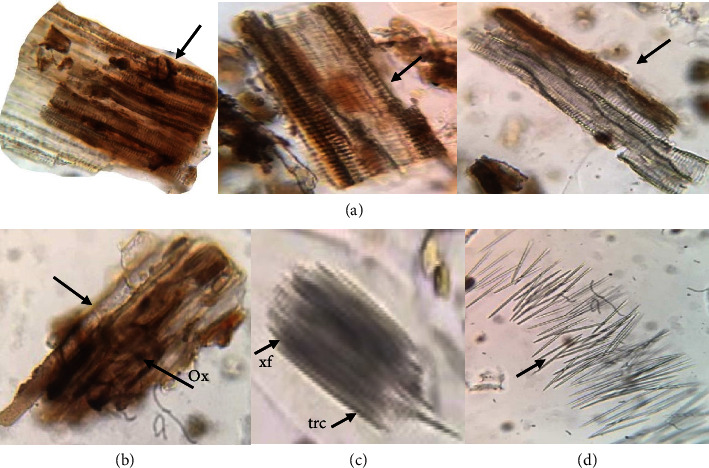
Microscopic analysis of the root organ powder from *C. latifolia* plants. Fragments of lignified xylem vessels with mesh and helical thickening (a), parenchyma with pith radius and calcium oxalate crystals (b), xylem fibers with tracheids (c), and trichomes (d). *Note*. Ox = oxalate crystal, xf = xylem fibers, and trc = tracheids.

Microscopic analysis was performed to provide the standardization parameters of *C. latifolia* plant organs (Figures 2–4). Microscopic analysis was performed using a binocular microscope at 400x magnification with chloral hydrate as the staining reagent. Chloral hydrate reagent is known to clarify preparations and dissolve cell contents for easier observation under a microscope.

Fragments (Figure 2) of the lower (Figure 2(A)) and upper (Figure 2(B)) epidermis accompanied by stomata were identified in the leaf organ powder. The lower epidermal fragment was the epidermis parallel to the low anomocytic-shaped stoma and was accompanied by a trichome (Figure 2(D)). The upper epidermis fragment (Figure 2(B)) showed a small number of stomata and parasitic forms. The epidermal fragment was also in direct contact with the cuticle layer, reducing the transpiration process. Microscopic analysis revealed fragments of sclerenchyma fibers (Figure 2(C)) in large amounts in the leaf organs of *C. latifolia*. Many sclerenchyma fibers indicate the leaf type of *C. latifolia*, which is a stiff leaf type. Other fragments found in leaf organs were bundle sheaths/vessels (Figure 2(E)) and fragments of pericyclic fibers with a tortuous wall (Figure 2(F)).

Figure 3 shows a microscopic form of the stem organ of the *C. latifolia* plant. The fragments identified were trichomes (Figure 3(A)) containing glands with unicellular head stalks. The stem organ powder of *C. latifolia* contained xylem vessel fragments thickened into a helical and annular vessel containing fibers (Figures 3(B)–(D)). The fragment of the periderm (Figures 3(E and F)) was part of the different covering tissues than the epidermis in the stem organ. This fragment serves to protect/cover the underlying tissue. The periderm replaces its position when the epidermis is pushed outward, damaged, and peeled off. The fragments identified showed that the stem organs in *C. latifolia* plants underwent secondary growth. Other fragments found in *C. latifolia* stem organs were endosperm containing oil droplets and aleurone grains containing calcium oxalate crystals (Figure 3(G)). This property is also found in many plants.

Microscopic analysis of *C. latifolia* plant root organ powder identified four fragments of xylem vessels, parenchymal cell fragments, xylem fibers, and trichomes (Figure 4). The network and helical thickening of the xylem vascular fragments were visualized (Figure 4(a)). This section also illustrates the presence of xylem fibers (Figure 4(c)) shaped like long cells with pointed ends. Tracheids were found in the longest cells, but at microscopic magnification, the shape of the tracheids cannot be seen in the xylem fiber fragments. Parenchymal cells (Figure 4(b)) with potassium oxalate crystals were also seen. The form of the potassium oxalate found was not determined microscopically. Another characteristic feature identified is the presence of unicellular trichomes and the absence of glands (Figure 4(d)).

### 3.2. Phytochemical Profile

The profile of phytochemical compounds from each *C. latifolia* plant organ extract was identified based on colorimetry with specific reagents.  Table 2 shows the group of compounds from each extract after spraying using staining reagents.

The results in Table 2 show that all extracts of each organ of the *C. latifolia* plant generally contain a group of phenolic compounds. Similar results were also found in identifying groups of flavonoid compounds. Flavonoid compounds have been found in ethanol, ethyl acetate, and hexane extracts from stem and leaf organs. Meanwhile, in the root organ, the flavonoid group was present only in the ethanol and ethyl acetate extracts, but no results were found in the hexane extract. Generally, positive results were found in identifying steroid/terpenoid groups in all organ extracts except leaf hexane and root ethanolic extracts. This group of saponin compounds is found only in the ethanolic extract of roots and stems. The presence of a group of alkaloid compounds with Mayer's and Dragendorff's reagents was found faintly in each extract, while with Wagner's reagent, it was found only in hexane and ethanol extracts of leaves and stems.

### 3.3. TPC and TFC Analysis

The TPC of the root, stem, and leaf extracts of *C. latifolia* plant organs was determined based on the gallic acid standard curve equation. The gallic acid concentration was varied to obtain a standard curve equation with a linear regression value of (*R*^2^) 0.9942 (*y* = 0.093 *x* + 0.0138). The results can be seen in Table 3.

The ethanol extracts gave the TPC in the order of root (68.63 ± 2.97 mg GAE/g), followed by the stem (65.95 ± 3.21 mg GAE/g) and leaves (13.59 ± 0.23 mg GAE/g). A similar trend was observed in the ethyl acetate and hexane extracts, where the root phenol levels were the highest at 64.1 ± 1.19 and 6.35 ± 0.18 mg GAE/g, respectively. Statistically, the TPC of the ethanol extract of the roots was not significantly different from the ethyl acetate extract of the stems and roots (*p*  >  0.05, *n* = 3). These results correlate well with the phytochemical profile data in Table 2, which shows the phenolic content of the ethanolic extract of roots and stems with a blue intensity.

tTable 3 shows the TFC of each plant organ of *C. latifolia*. The ethyl acetate extract of the stems yielded the highest TFC content with 14.33 ± 0.71 mg QE/g, followed by the roots and leaves, 10.35 ± 0.08 and 3.92 ± 0.45 mg QE/g, respectively. Among the ethanol extracts, the leaves had the highest TFC values of 10.21 ± 0.23 mg QE/g, followed by the stems and the roots with TFC values of 7.71 ± 0.15 and 7.44 ± 0.15 mg QE/g, respectively. Meanwhile, the hexane extract of the leaves revealed the highest content of TFC (9.81 ± 0.14 mg QE/g) compared to other organs. Statistically, the ethyl acetate extract of the stems had the highest TFC values and was significantly different among all extracts (*P* < 0.05, *n* = 3, post hoc test LSD).

### 3.4. Antioxidant Evaluation of *C. latifolia* Extract

Evaluation of the antioxidant activity of *C. latifolia* plant organ extracts aimed at investigating the ability of antioxidant compounds in each extract to scavenge free radicals (DPPH and NO scavenging assays) and reduce metal ions (CUPRAC assay). The antioxidant activities were described as IC_50_ values and *µ*mol GAEAC/g extract (Table 4). Based on categorization by Blois [34], the antioxidant power in reducing free radicals was categorized as extreme activity (IC_50_ < 50 *µ*g/mL), strong activity (50–100 *µ*g/mL), moderate activity (>100–150 *µ*g/mL), and weak activity (>150 *µ*g/mL).

The NO• scavenging activities of the leaf extracts (hexane, ethyl acetate, and ethanol) were stronger than those of the stems and roots. IC_50_ values of 88.38 ± 0.29 *µ*g/mL (categorized as strong), 25.51 ± 0.58 *µ*g/mL (extreme category), and 22.57 ± 0.74 *µ*g/mL (extreme category) were obtained for hexane, ethyl acetate, and ethanol extracts, respectively. The root extracts showed a different trend for each extract, with the ethanol extract (84.60 ± 0.56 *µ*g/mL) having a strong potential and ethyl acetate (123.47 ± 0.52 *µ*g/mL) having a moderate potential for reducing NO• radicals. In contrast, the hexane extract of the root organ shows a weak potential with an IC_50_ value of 372.65 ± 0.58 *µ*g/mL. The potential antioxidant activities revealed significantly different effects (*p* < 0.05, post hoc test, LSD) for each extract from different plant organs. The total NO test showed that the ethanol and ethyl acetate extracts of the leaf organs and the ethanol extract of the stems showed extreme activity compared to other extracts. The ethyl acetate and ethanol extracts from leaf organs had the same category of quercetin antioxidant activity as a positive control (3.59 ± 0.072 *µ*g/mL), which was very potent. Previous studies have not reported the antioxidant potential of each extract and organ of the *C. latifolia* plant in reducing NO• radicals. Therefore, the results obtained cannot be compared.

The DPPH• scavenging activities of the plant organs showed a slightly different trend than the NO• scavenging activity. Extreme scavenging activities were observed for the ethanolic root and stem extracts and the root ethyl acetate extract with IC_50_ values of 20.42 ± 0.33, 41.19 ± 0.32, and 28.30 ± 0.55 *µ*g/mL, respectively, with the activity category (extreme category) of the extracts being the same as that of the positive control (4.76 ± 0.09 *µ*g/mL). The ethanol extract of the leaves and the ethyl acetate extracts of the stems and leaves showed potent activity with IC_50_ values of 50–100 *µ*g/mL. In contrast, hexane extracts from leaves, stems, and roots showed a weak effect in reducing DPPH• radicals (IC_50_ > 200 *µ*g/mL).

Unlike the NO• and DPPH• scavenging methods, the antioxidant potential in the CUPRAC method is based on the ability of antioxidant compounds in the sample to reduce Cu^2+^ ions to Cu ^+^ ions. The magnitude of the reducing power corresponds to that of gallic acid. The greater the reducing power, the stronger the antioxidant potential in a sample. The reducing power of each extract is shown in Table 4. All ethyl acetate extracts from each organ showed higher reducing power, followed by ethanol extracts from each organ of the *C. latifolia* plant. All plant organs found the lowest reducing power in the hexane extract samples. Significant intersample antioxidant activity was detected in the ethyl acetate extract (390.42 ± 14.49 *µ*M/g extract) of *C. latifolia* leaves (*p* < 0.05, post hoc test, LSD, *n* = 3). The antioxidant potential of ethyl acetate leaf extract in the CUPRAC method correlated with the antioxidant potential of the ethyl acetate leaf extract with an extreme category of the NO• radical scavenger method. To date, no study has reported yet on the antioxidant activity of extracts from the leaves, stems, and roots of the *C. latifolia* plant, including the radical scavenging and metal ion reduction activities.

### 3.5. Antityrosinase Evaluation of *C. latifolia* Extract

Root ethanol extract had the lowest IC_50_ value of 108.5 *µ*g/mL, indicating strong tyrosinase inhibitory activity compared to other samples. However, the hexane extracts of leaves, stems, and roots showed no tyrosinase inhibitory activity with IC_50_ values at concentrations >1000 *µ*g/mL (IC_50_ values obtained from exploration data). The ethanol extracts of the stems and leaves and the ethyl acetate root extract showed moderate inhibitory activity. IC_50_ values <100 *µ*g/mL indicated a strong inhibitory potential, IC_50_ values of 100–450 *µ*g/mL a moderate inhibitory potential, and IC_50_ values of 450–700 *µ*g/mL a weak inhibitory potential. Kojic acid as a positive control achieved an IC_50_ value of 5.55 *µ*g/mL with a strong potential compared to *C. latifolia* root ethanolic extract. However, the ethanolic extract of *C. latifolia* root still has a strong potential to inhibit tyrosinase activity, so it can be considered a candidate for skin lightening (depigmentation).

### 3.6. The Phenolic Composition of the Active Extract by LC-ESI-MS Study

Extracts from *C. latifolia*, which were found to have moderate to strong antioxidant and antityrosinase activities in vitro, were assayed for phenolic compound content. Therefore, the ethyl acetate root extract and the ethanol extract of the roots, stems, and leaves were analysed for their phenolic compounds using the LC-ESI-MS method.  Table 5 shows the group of phenolic compounds identified based on LC-ESI-MS analysis.

**Figure 5:**
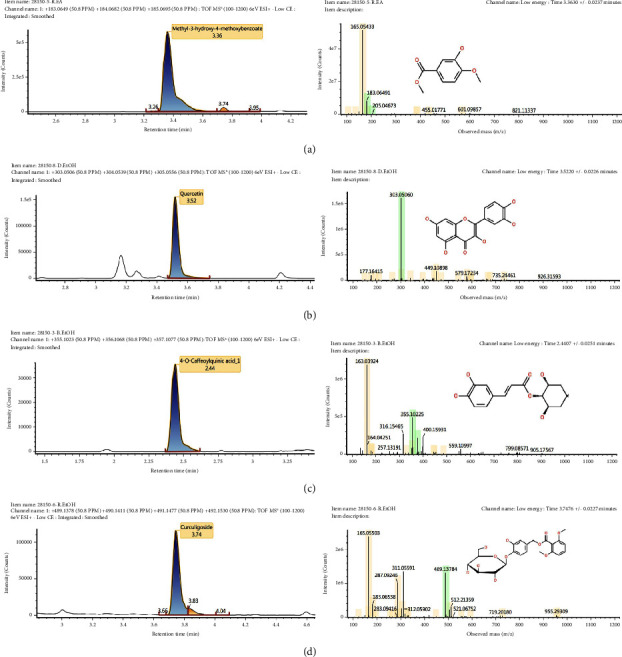
Phenolic compound chromatograms and mass spectrum of the ethyl acetate root extract (a), ethanol leaf extract (b), ethanol stem extract (c), and ethanol root extract (d).

In each extract, the presence of phenolic compounds in the form of methyl 3-hydroxy-4-methoxybenzoate (183.06 *m*/*z*), quercetin (303.05 *m*/*z*), 4-O-caffeoylquinic acid-1 (355, 10 *m*/*z*), and curculigoside (489.14 *m*/*z*) was identified in the ethyl acetate root extract and the ethanol extract from leaves, stems, and roots, respectively (Figure 5). Quercetin and curculigoside were also identified in the ethanolic extract of *C. latifolia* leaves and roots by the study of Zolghadri et al. [3] using the UHPLC-Q-Orbitrap-HRMS approach. However, the compounds methyl 3-hydroxy-4-methoxybenzoate and 4-O-caffeoylquinic acid-1 identified in the ethyl acetate root extract and the ethanol stem extract provide additional information regarding the class of phenolic compounds present in *C. latifolia*.

## 4. Discussion

This study tested the antioxidant and antityrosinase activities of *C. latifolia* extracts. This study also provides supporting information on the microscopic properties and screening of phytocomponents from each plant organ extract to supplement the scientific data on these plants. Although previous studies have reported on the phytochemical screening and antioxidant evaluation, the available information is limited to one part of the plant. Hence, the current research has provided more thorough information on various plant organs, including roots, stems, and leaves. In addition, this study also examined the phytochemical and bioactivity profiles of several extracts using solvents such as *n*-hexane, ethyl acetate, and ethanol-aqueous (70% v/v). Previous studies examining *C. latifolia* have primarily focused on its bioactivity as an antidiabetic and antibacterial agent. The present work is the first to report on the antioxidant and antityrosinase activities and the association between these bioactivities.

The reported organoleptic and microscopic properties of *C. latifolia* are part of the standardization parameters for raw materials. Organoleptic and microscopic assessments identify the purity, safety, and quality assurance of natural raw materials to ensure that these raw materials are not added to synthetic raw materials when used as standardized herbal medicinal products [22, 35]. The organoleptic evaluation of each plant organ was based on sensory analysis to identify shape, color, odour, and taste (Table 1). Microscopic analysis of the dry powder of each plant organ revealed differences in each fragment. Some of the identified fragments have their uniqueness. Therefore, the results of the microscopic analysis can provide an overview of the characteristics of *C. latifolia* plants that have not yet been reported. The results of an organoleptic and microscopic analysis can help to assess the purity of natural medicinal products [36]. In phytochemical screening studies, *C. latifolia* extract has been shown to generally contain phenolic, flavonoid, and steroid/terpenoid compounds (Table 2). Several studies have shown that the roots and leaves of *C. latifolia* contain phenolic glycoside compounds such as curculigoside, orcinol glycoside, phloridzin, pomiferin, scandenin, and mundulon [3, 5]. In addition, the roots and leaves also showed the presence of steroid/terpenoid compounds such as curculigosaponin and cycloartane triterpene [10]. The present study confirmed the previous reports. It is worth noting that no chemical analysis of the stem organs of *C. latifolia* has been reported. The presence of *C. latifolia* chemical compounds can provide a further understanding of their pharmacological bioactivities.

Plant organs may contain different phenolic compounds [37–39]. However, the extraction solvent affects the ability to extract different phenols from each plant organ. In general, this study showed that the root organs of *C. latifolia* had higher phenolic content than other organs (Table 3). This study also adds to the data that ethanol solvents can extract phenolic compounds. Compared to other solvents, ethanol can extract the most extensive phenolic compounds. The high polarity of phenols results in a more effective extraction of phenols by polar solvents such as ethanol than other solvents [40].

Similarly, studies by Umar et al. [10] showed that the ethanolic extract of the roots provided high levels of phenols compared to the ethanolic extract of the leaves. Determination of TPC in the stem organs of *C. latifolia* plants has not been reported. Therefore, this study complemented the data related to TPC in other organs of *C. latifolia* plants. In the TFC test, *C. latifolia* extract was reported to have different concentrations in each organ. The different profiles of flavonoid content indicate that each part of the plant has a different accumulation of flavonoid content. Studies by Umar et al. [10] also reported similar results that the leaf organ ethanol extract had a higher TFC than the root ethanol extract. However, no data on the TFC of the ethyl acetate and hexane extract of *C. latifolia* have been reported. The results of the present study can provide an overview of comparisons of TFC levels using different solvents and plant organs of *C. latifolia*.

A plant's high levels of total phenols and flavonoids also offer great potential for its antioxidant activity. *C. latifolia* plants are supported by high levels of phenols and flavonoids in every part of the plant. Flavonoids, the main phenolic group, play a role in biological activity, including their great activity in reducing various free radicals. The more hydroxy groups attached to the phenolic aromatic nucleus provide greater reducing power towards free radicals. Several previous studies have shown that the antioxidant activity is increased due to the presence of phenolic hydroxyl in the A and B rings of the flavonoid skeleton [41, 42]. There are several reasons that the more phenolic hydroxyl groups present in a compound, the greater the antioxidant effect. For several reasons, the more hydroxyl groups, the more hydrogen is donated to free radicals to stabilize the radical reaction. The phenolic hydroxyl group exerts a strong electronic effect on the radicals, making it easier to neutralize the radical reactions [19, 43].

The presence of phenolic and flavonoid compounds from *C. latifolia* plants supports the antioxidant activity. The antioxidant activity in this study showed that the ethanol and ethyl acetate extracts from each organ of the *C. latifolia* plant tended to provide strong to very strong antioxidant activity as tested by three different test methods, including DPPH, NO, and CUPRAC assays. Testing the bioactivity of *C. latifolia* extract as an antioxidant tended to give the same results across the three methods used in this study. One or two types of extracts that do not correlate with the test results of the three methods used may be affected by the chemical content of each extract in terms of content and compound group. Several studies relevant to the potential of *C. latifolia* to reduce DPPH radicals showed that ethanol and aqueous extracts from plant roots were the most potent antioxidants [9, 10, 12, 33]. In the ethanol extract of leaf organs, the present study showed a strong activity in reducing DPPH.

In contrast, results found in the study of [10, 33] showed weak potential for reducing DPPH radicals by the ethanol extract of leaf organs. However, no information has been reported on the bioactivity of stem organs and extracts of ethyl acetate and hexane from *C. latifolia* plant organs. Testing of the antioxidant bioactivity of *C. latifolia* plants using the NO and CUPRAC methods is still limited, so the informational data obtained can be used as a reference for future research developments. Antioxidant activity testing using DPPH and NO radical scavenging methods and Cu reduction aimed at observing the antioxidant bioactivity profile of compounds from *C. latifolia* plants. DPPH and NO are free radicals that can be converted by biochemical reactions in the body to modulate the formation of reactive oxygen-nitrogen species (RONS). The formation of RONS can be accelerated by a catalytic reaction in the presence of metals such as copper, leading to a transfer of copper ions that generates oxidative stress [44, 45]. There was a biochemical mechanism for the formation of RONS,, so the three methods were carried out to find out the description of the bioactivity of *C. latifolia* compounds as antioxidants.

Several studies suggest that excess reactive species, including reactive oxygen species and reactive nitrogen species, aggravate pigmentation, which in turn causes uneven skin tone, pigmentation disorders, roughness, and even aging. In the present study, tyrosinase inhibitory activity was determined to assess the potential of *C. latifolia* plant organs to inhibit melanin formation when used as a whitening agent in the cosmetics industry [11, 35]. Mushroom tyrosinase catalyzes the formation of L-tyrosine from L-DOPA, which subsequently forms the dopachrome. When adding an inhibitor in the form of *C. latifolia* extract, the color of the solution faded due to the inhibitory activity of the tyrosinase enzyme [30, 46].  Table 6 shows the antityrosinase activities of each *C. latifolia* organ extract. In vitro, the ethyl acetate extract of the roots and the ethanol extract of the stems and leaves had moderate tyrosinase inhibitory activity. In contrast, the ethanol extract of the roots of *C. latifolia* showed potent tyrosinase inhibitory activity.

Nonetheless, the kojic acid positive control activity was significant, giving it the strongest activity among the extracts. However, it can still be assumed that *C. latifolia* extract contains compounds that inhibit tyrosinase activity. The tyrosinase inhibitory activities of root organ ethanol extract were supported by the presence of phenolic and flavonoid compounds (Tables 2 and 5), which are responsible for the reduction of tyrosinase activity. Phenol and flavonoid derivatives (Figure 5), including methyl 3-hydroxy-4-methoxybenzoate, quercetin, 4-O-caffeoylquinicacid-1, and curculigoside, can act as tyrosinase inhibitors [3]. Hydroxyl groups in rings A and B of the flavonoid compounds have inhibitory activity by directly binding to tyrosinase and Cu^2+^ chelates as metalloenzymes in tyrosinase. In addition, it is predicted that the formation of the Cu^2+^ complex of tyrosinase with the catechol structure (3,4-) of the dihydroxy group on the C-ring of quercetin also affects the inhibition of tyrosinase [46–49]. The bioactivity of *C. latifolia* extract provides new information as an antityrosinase that has not been previously reported.

**Figure 6:**
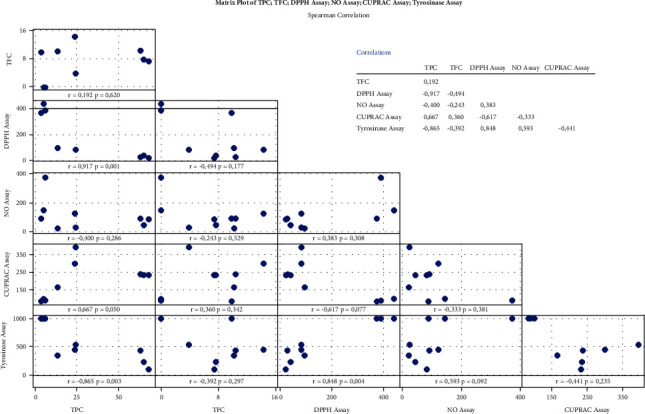
Spearman's correlation coefficient analysis between TPC and TFC with antioxidant and antityrosinase activities of nine extracts in triplicate. *p* value <0.01 indicates variable significance. TPC: total phenolic content and TFC: total flavonoid content.

A correlation analysis, adopted from Spearman's method, was performed to ensure a correlation between the research results obtained. Spearman's correlation aims at expressing the strength and direction of the linear correlation between TPC and TFC on antioxidant and antityrosinase activities. The levels of phenols and flavonoids (mg/g extract, *n* = 3) of each extract were correlated with the IC_50_ value (*n* = 3) of the antioxidant and antityrosinase activities of each extract (Figure 6). The correlation coefficient plot matrix showed different correlations. The strongest category correlation between TPC and antioxidant activity was found in the DPPH method with *r* = −0.917, *p* < 0.01. These results show that the antioxidant activity of each extract is influenced by 91.7% by the presence of phenolic compounds, while other compounds influence only 8.3%. The correlation coefficient of TPC with an antioxidant activity using the CUPRAC and NO scavenger methods was categorized as strong (*r* = 0.667) and moderate (*r* = 0.400), respectively.

In contrast, the matrix plot between TFC and antioxidant activity showed weak to moderate correlations. In the DPPH radical reduction method, the correlation of its activity was obtained due to the influence of flavonoid compounds with a medium category (*r* = 0.494). At the same time, the antioxidant activity of the NO and CUPRAC methods revealed a weak correlation with flavonoid compounds (Figure 6).

The antityrosinase activity of each extract also correlated with the content of phenolic compounds in each sample with a correlation coefficient of *r* = 0.865 (*p* < 0.01), while the other compounds affected the rest. However, the presence of flavonoid compounds in each extract resulted in a weak correlation coefficient (*r* = 0.392, *p* > 0.01) for its antityrosinase activity (Figure 6). However, this study shows that the major compounds in phenols were predicted to contribute to their bioactivity as antioxidants and antityrosinases.

## 5. Conclusions

This study reported the antioxidant and tyrosinase inhibitory activities of several extracts of the *C. latifolia* plant. Microscopic studies and the correlation between TPC and TFC with antioxidant and antityrosinase bioactivities were reported for the first time. It was concluded that the ethanol extracts from the roots, stems, and leaves of *C. latifolia* provide potent antioxidant and antityrosinase activities and are supported by high levels of total phenolic compounds and flavonoids. The LC-ESI-MS identified phenolic compounds from *C. latifolia* plants. Information obtained from the present study could not yet describe the actual mechanisms for the activity of antityrosinase. Therefore, an in-depth study of this mechanism is still needed, both in silico and in vivo. We hope that the results of our study provide scientific information for the development of *C. latifolia* plants as candidate skin-lightening ingredients and, in the future, can find markers that can be used as candidates for effective skin-lightening agents and formulated into cosmetic dosage forms.

## Figures and Tables

**Table 1:** Organoleptic evaluation of organ plants from *C. latifolia*.

Parameter	Plant organ of *C. latifolia*		
	Root	Stem	Leaves
Shape	Long round	Triangular, elongated, and smooth surface	Linear
Color	Brown	Brownish green	Dark green
Odour	Aromatic (distinctive odour)	Aromatic (distinctive odour)	Aromatic (distinctive odour)
Taste	Bitter	No taste	No taste

**Table 2:** Phytochemical profile of each extract by specific reagent.

Organ	Extract	Group of compounds						
		Phenolic	Flavonoid	Saponin	Steroid/terpenoid	Alkaloid		
						Mayer	Dragendorff	Wagner
Leaves	Hexane extract	+	+	—	—	—	—	+
	Ethyl acetate extract	+	+	—	+	—	—	—
	Ethanol extract	+	+	—	+	—	—	+

Stems	Hexane extract	+	+	—	+	—	—	+
	Ethyl acetate extract	+	+	—	+	—	—	—
	Ethanol extract	+	+	+	+	—	—	+

Roots	Hexane extract	+	—	—	+	—	—	—
	Ethyl acetate extract	+	+	—	+	—	—	—
	Ethanol extract	+	+	+	—	—	—	—

*Note*. “—” indicates the absence of compounds and “+” indicates the presence of compounds.

**Table 3:** TPC and TFC analysis of roots, stems, and leaves in *n*-hexane, ethyl acetate, and ethanol (96% v/v) extracts of *C. latifolia*.

Sample	GAE (TPC) (mg/g extract)	QE (TFC) (mg/g extract)
Hexane leaf extract	3.66 ± 0.13^*∗*^^b^	9.81 ± 0.14^*∗*^^g^
Hexane stem extract	5.19 ± 0.23^*∗*^^ac^	0.49 ± 0.01^*∗*^^c^
Hexane root extract	6.35 ± 0.18^*∗*^^b^	0.45 ± 0.01^*∗*^^a^
Ethyl acetate leaf extract	24.76 ± 0.19^*∗*^^e^	3.92 ± 0.45^*∗*^
Ethyl acetate stem extract	24.45 ± 0.32^*∗*^^d^	14.33 ± 0.71^*∗*^
Ethyl acetate root extract	64.1 ± 1.19^*∗*^^h^	10.35 ± 0.08^*∗*^^g^
Ethanol leaf extract	13.59 ± 0.23^*∗*^	10.21 ± 0.23^*∗*^^af^
Ethanol stem extract	65.95 ± 3.21^*∗*^^fi^	7.71 ± 0.15^*∗*^^i^
Ethanol root extract	68.63 ± 2.97^*∗*^^h^	7.44 ± 0.15^*∗*^^h^

*Note.* ^*∗*^indicates a significant difference between samples (*p* < 0.05, *n* = 3, post hoc test LSD). Alphabet differences indicate no significant difference between samples (*p* > 0.05, *n* = 3, post hoc test LSD).

**Table 4:** Antioxidant activity of hexane, ethyl acetate, and ethanol extracts of the leaves, stems, and roots of *C. latifolia* plants.

Extract of *C. latifolia*	Inhibition concentration of 50% (*µ*g/mL)		*µ*mol GAEAC/g extract
	DPPH• assay	NO• assay	CUPRAC assay
Hexane leaf extract	370.74 ± 0.51^*∗*^	88.38 ± 0.29^*∗*^	87.17 ± 5.65^*∗*^^bc^
Hexane stem extract	437.07 ± 0.59^*∗*^	143.32 ± 0.31^*∗*^	102.52 ± 12.98^*∗*^^ac^
Hexane root extract	389.16 ± 1.81^*∗*^	372.65 ± 0.58^*∗*^	93.18 ± 6.2^*∗*^^ab^
Ethyl acetate leaf extract	82.01 ± 0.61^*∗*^^e^	25.51 ± 0.58^*∗*^	390.42 ± 14.49^*∗*^
Ethyl acetate stem extract	81.22 ± 0.43^*∗*^^d^	123.47 ± 0.52^*∗*^	298.61 ± 3.05^*∗*^
Ethyl acetate root extract	28.30 ± 0.55^*∗*^	92.08 ± 0.65^*∗*^	236.29 ± 5.01^*∗*^^hi^
Ethanol leaf extract	94.85 ± 0.52^*∗*^	22.57 ± 0.74^*∗*^	165.01 ± 1.88^*∗*^
Ethanol stem extract	41.19 ± 0.32^*∗*^	45.65 ± 0.77^*∗*^	234.58 ± 14.17^*∗*^^fi^
Ethanol root extract	20.42 ± 0.33^*∗*^	84.60 ± 0.56^*∗*^	232.08 ± 5.61^*∗*^^fh^
Quercetin	4.76 ± 0.09^*∗*^	3.59 ± 0.072^*∗*^	—

*Note.* ^*∗*^Significant difference between samples (*p* < 0.05, *n* = 3, post hoc test LSD). Alphabet differences indicate no significant difference between samples (*p* > 0.05, *n* = 3, post hoc test LSD).

**Table 5:** LC-ESI-MS profile of active extract *C. latifolia*.

No.	Extract	RT (min)	Observed MS (*m*/*z*)	Compounds	Molecular formula
1	EA root extract	3.36	183.06	Methyl-3-hydroxy-4-methoxybenzoate	C_9_H_10_O_4_
2	Ethanol leaf extract	3.52	303.05	Quercetin	C_15_H_10_O_7_
3	Ethanol stem extract	2.44	355.10	4-O-caffeoylquinic acid-1	C_16_H_18_O_9_
4	Ethanol root extract	3.75	489.14	Curculigoside	C_22_H_26_O_11_

**Table 6:** Evaluation of antityrosinase activity of hexane, ethyl acetate, and ethanol extracts of the leaves, stems, and roots of *C. latifolia* plants.

Extract	Concentration (*µ*g/mL)							IC_50_ (*µ*g/mL)	Activity
	6.25	12.5	25	50	100	200	400		
Hexane leaf extract	—	16.64 ± 0.61	19.32 ± 0.58	19.72 ± 0.47	23.82 ± 3.53	26.63 ± 1.22	3193 ± 0.00	>1000^*β*^^*∗*^^bc^	Inactive
Hexane stem extract	—	18.48 ± 1.01	22.11 ± 1.24	23.63 ± 2,86	27.14 ± 0.66	30.63 ± 0.49	36.30 ± 1.01	>1000^*β*^^*∗*^^ac^	Inactive
Hexane root extract	—	17.63 ± 7.72	21.48 ± 0.50	22.6.7 ± 4.20	24.69 ± 0.19	27.03 ± 0.58	33.37 ± 1.83	>1000^*β*^^*∗*^^ab^	Inactive
EA leaf extract	16.24 ± 0.60	20.05 ± 0.51	22.47 ± 0.46	24.92 ± 3.48	29.13 ± 1.36	32.91 ± 0.00	—	534.2^*β*^^*∗*^	Weak
EA stem extract	22.84 ± 1.81	23.92 ± 1.87	24.72 ± 2.81	27.24 ± 1.00	31.63 ± 0.48	37.91 ± 0.33	—	454.2^*β*^^*∗*^^f^	Weak
EA root extract	21.92 ± 0.89	23.18 ± 0.09	23.09 ± 2.01	23.65 ± 1.03	28.08 ± 0.58	34.33 ± 1.80	—	434.1^*β*^^*∗*^^d^	Moderate
Ethanol leaf extract	—	34.89 ± 1.62	38.41 ± 0.84	41.47 ± 0.92	43.84 ± 2.80	46.93 ± 2.40	51.20 ± 1.92	349.5^*∗*^	Moderate
Ethanol stem extract	—	31.63 ± 1.76	34.85 ± 3.81	38.72 ± 4.08	43.99 ± 2.63	48.42 ± 2.12	54.10 ± 2.11	242.6^*∗*^	Moderate
Ethanol root extract	—	34.17 ± 0.27	36.99 ± 2.67	42.50 ± 2.28	45.73 ± 1.30	53.56 ± 0.92	66.03 ± 0.40	108.5^*∗*^	Strong
Kojic acid	IC_50_ (5.55 *µ*g/mL)^*∗*^								Robust

*Note*. ^*∗*^indicates a significant difference between samples in rows (*p* < 0.05, *n* = 3, post hoc test, LSD). Alphabet differences indicate no significant difference between samples in rows (*p* > 0.05, *n* = 3, post hoc test LSD). ^*β*^The IC_50_ value was determined by data exploration. EA: ethyl acetate.

## Data Availability

The data used to support the findings of this study are available from the corresponding author upon request.
